# Genome-Wide Identification and Expressional Analysis of the *TIFY* Gene Family in *Eucalyptus grandis*

**DOI:** 10.3390/ijms26167914

**Published:** 2025-08-16

**Authors:** Chunxia Lei, Yingtong Huang, Rui An, Chunjie Fan, Sufang Zhang, Aimin Wu, Yue Jing

**Affiliations:** 1 Guangdong Key Laboratory for Innovative Development and Utilization of Forest Plant Germplasm, College of Forestry and Landscape Architecture, South China Agricultural University, Guangzhou 510642, China; cxlei@stu.scau.edu.cn (C.L.); 202218130110@stu.scau.edu.cn (Y.H.); ruian@stu.scau.edu.cn (R.A.); sufangzhang@henau.edu.cn (S.Z.); wuaimin@scau.edu.cn (A.W.); 2 State Key Laboratory of Tree Genetics and Breeding, Key Laboratory of State Forestry and Grassland Administration on Tropical Forestry, Research Institute of Tropical Forestry, Chinese Academy of Forestry, Guangzhou 510520, China; fanchunjie@caf.ac.cn; 3 Guangdong Provincial Key Laboratory of Utilization and Conservation of Food and Medicinal Resources in Northern Region, Shaoguan University, Shaoguan 512005, China

**Keywords:** *TIFY* family genes, expression analysis, functional analysis, *Eucalyptus grandis*

## Abstract

The *TIFY* gene family participates in crucial processes including plant development, stress adaptation, and hormonal signaling cascades. While the *TIFY* gene family has been extensively characterized in model plant systems and agricultural crops, its functional role in *Eucalyptus grandis*, a commercially valuable tree species of significant ecological and economic importance, remains largely unexplored. In the present investigation, systematic identification and characterization of the *TIFY* gene family were performed in *E. grandis* using a combination of genome-wide bioinformatics approaches and RNA-seq-based expression profiling. Nineteen *EgTIFY* genes were identified in total and further grouped into four distinct subfamilies, TIFY, JAZ (subdivided into JAZ I and JAZ II), PPD, and ZML, based on phylogenetic relationships. These genes exhibited considerable variation in gene structure, chromosomal localization, and evolutionary divergence. Promoter analysis identified a multitude of cis-acting motifs involved in mediating hormone responsiveness and regulating abiotic stress responses. Transcriptomic profiling indicated that *EgJAZ9* was strongly upregulated under methyl jasmonate (JA) treatment, suggesting its involvement in JA signaling pathways. Taken together, these results offer valuable perspectives on the evolutionary traits and putative functional roles of *EgTIFY* genes.

## 1. Introduction

TIFY transcription factors, a plant-specific family first identified in *Arabidopsis thaliana*, belong to the GATA transcription factor superfamily and are characterized by a highly conserved TIFY domain encompassing the core motif TIF[F/Y]XG [1]. The molecular functions of TIFY proteins are primarily associated with protein–protein interactions and regulation of signal transduction, particularly within the jasmonic acid (JA) signaling pathway [2,3,4]. The presence of conserved domains underpins the division of the *TIFY* gene family into four main subfamilies: TIFY, JAZ (further divided into JAZ I and JAZ II), ZML, and PPD [5,6]. The TIFY subfamily is distinguished by the presence of solely the core TIFY domain [7]. The JAZ subfamily is distinguished by the co-occurrence of the TIFY domain and a C-terminal Jas domain, which is essential for JA signaling [8]. ZML subfamily members possess the TIFY domain, a GATA-type zinc finger domain, and a CCT (CONSTANS, CO-like, and TOC1) motif [9]. The PPD subfamily is characterized by the presence of a PPD domain, a TIFY domain, and a truncated Jas domain that lacks the conserved PY motif [10]. Such structural heterogeneity across subfamilies mirrors their functional divergence and emphasizes their importance in modulating plant growth and development, physiological activities, abiotic stress responses, and hormone signaling pathways.

*TIFY* gene families have been identified and characterized in a multitude of plant species, with 18 members documented in *A. thaliana* [3], 20 in *Oryza sativa* [11], 15 in *Salvia miltiorrhiza* [12], 19 in *Brachypodium distachyon* [13], 30 in maize (*Zea mays*) [14], 16 in *Capsicum annuum* [15], 21 in *Juglans regia* [16], 16 in *Camellia sinensis* [17], and 25 in *Populus trichocarpa* [7]. Functional studies in *A. thaliana* have established the participation of *TIFY* genes in regulating various developmental processes, such as root hair development, lateral root formation, stamen development, and leaf senescence [18,19]. Of these, the JAZ subfamily has garnered the most intensive scrutiny, attributable to its pivotal role in JA signaling pathways. JAZ proteins act as repressive regulators in jasmonate signaling through interactions with basic helix–loop–helix (bHLH) transcription factors such as MYC2 and MYC3, thereby negatively regulating jasmonate-mediated physiological responses [8,18,20]. Overexpression of *AtTIFY1*/*AtZIM* facilitates petiole and hypocotyl elongation via the induction of longitudinal cellular expansion [3]. ZML subfamily members have been implicated in regulating hormone pathways related to photoperiodic responses, while *AtTIFY4a* and *AtTIFY4b* of the PPD subfamily have been shown to control leaf size and margin curvature in *A. thaliana* [21,22].

*E. grandis*, a globally significant fast-growing tree species, holds high economic, ecological, and industrial value. It is widely cultivated for its desirable wood properties and serves as a key raw material in industries such as paper production, construction, and furniture manufacturing [23]. Given its commercial importance and broad adaptability, *E. grandis* has emerged as a model species for forest genomics research. However, despite the established significance of *TIFY* genes in plant physiological processes, their systematic characterization in *E. grandis* remains unreported.

In the current study, a genome-wide identification and analysis of the *TIFY* gene family in *E. grandis* were performed through an integration of genome-wide bioinformatics methodologies and RNA-seq-driven expression profiling. These genes were analyzed for their physicochemical properties, conserved motifs, chromosomal distribution, and expression profiles. The expression levels of *JAZ* subfamily members, including *EgJAZ9*, *EgJAZ10*, and *EgJAZ11*, were significantly upregulated following JA treatment. Their protein sequences contain highly conserved Jas domains similar to those found in *Arabidopsis* JAZ proteins, indicating their potential involvement in jasmonate signaling. Among them, *EgJAZ9* emerges as a promising candidate for further functional investigation. Our results offer valuable perspectives on the structural features and putative functions of *EgTIFY* genes, laying a foundation for future functional studies and contributing to a deeper understanding of the *TIFY* family’s role in *Eucalyptus* development and stress responses.

## 2. Results

### 2.1. Identification and Physicochemical Characteristics of EgTIFY Proteins

TIFY protein sequences derived from *A. thaliana* were employed as query sequences in a BLASTp (TBtools-II) analysis to conduct comparative sequence alignments with *E. grandis* protein sequences. Based on sequence homology and conserved domain analyses, a total of 19 TIFY family members were identified in *E. grandis* and categorized into four distinct subfamilies: JAZ (subdivided into JAZ I and JAZ II), PPD, TIFY, and ZML (Table 1). Physicochemical analyses revealed notable differences among the EgTIFY proteins in terms of sequence length, instability index, theoretical isoelectric point (pI), and molecular weight. The proteins varied in length from 104 to 443 amino acids, with EgTIFY1 representing the largest (443 aa) and EgJAZ9 the smallest (104 aa).

Isoelectric point analysis indicated that 13 EgTIFY proteins have pI values above 7, while 6 have values below 7, suggesting that the majority of these proteins are basic in nature. The aliphatic index, which reflects thermostability, ranged from 31.19 (EgZML1) to 83.27 (EgJAZ5), indicating relatively minor variation in thermal stability among the family members. All EgTIFY proteins displayed negative GRAVY (Grand Average of Hydropathy) scores, confirming their hydrophilic nature. Among them, EgZML3 was the most hydrophilic (GRAVY = −0.57), while EgJAZ12 showed the lowest degree of hydrophilicity (GRAVY = −0.31). Subcellular localization predictions indicated that all 19 EgTIFY proteins are presumably localized within the nucleus (Table 1).

### 2.2. Phylogenetic Tree of EgTIFYs

**Figure 1:**
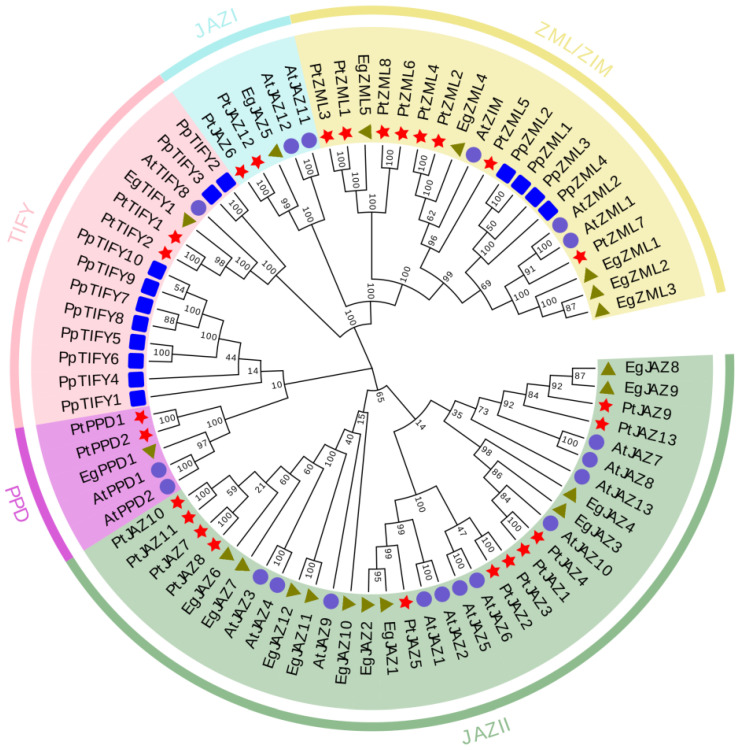
Phylogenetic tree of *TIFY* genes in *E. grandis*, *P. trichocarpa*, *A. thaliana,* and *Physcomitrium patens*. Symbols of different shapes represent *TIFY* genes from *E. grandis* (green triangles, *Eg*), *A. thaliana* (blue circles, *At*), *P. trichocarpa* (red stars, *Pt*), and *P. patens* standing bowl moss (blue squares, *Pp*). The neighbor-joining tree was constructed with 1000 bootstrap replicates.

To dissect the evolutionary associations of the *TIFY* gene family across plant lineages, a phylogenetic tree was constructed utilizing full-length TIFY protein sequences originating from *E. grandis* (19 sequences), *A. thaliana* (19), *Populus trichocarpa* (25), and *Physcomitrella patens* (14). Following sequence alignment, a neighbor-joining phylogenetic tree was generated (Figure 1). The phylogenetic tree delineated four major subfamilies: JAZ (encompassing JAZ I and JAZ II), PPD, TIFY, and ZML. Among these, the JAZ II subfamily contained the largest number of *EgTIFY* genes (11), whereas the JAZ I, PPD, and TIFY subfamilies each included a single member. The ZML subfamily comprised five *EgTIFY* genes. Notably, *TIFY* genes from *E. grandis* and *P. trichocarpa* were dispersed across multiple shared branches, signifying a relatively close evolutionary affinity between these two species (Figure 1).

### 2.3. Analysis of Gene Structures and Conserved Domains of EgTIFYs

**Figure 2:**
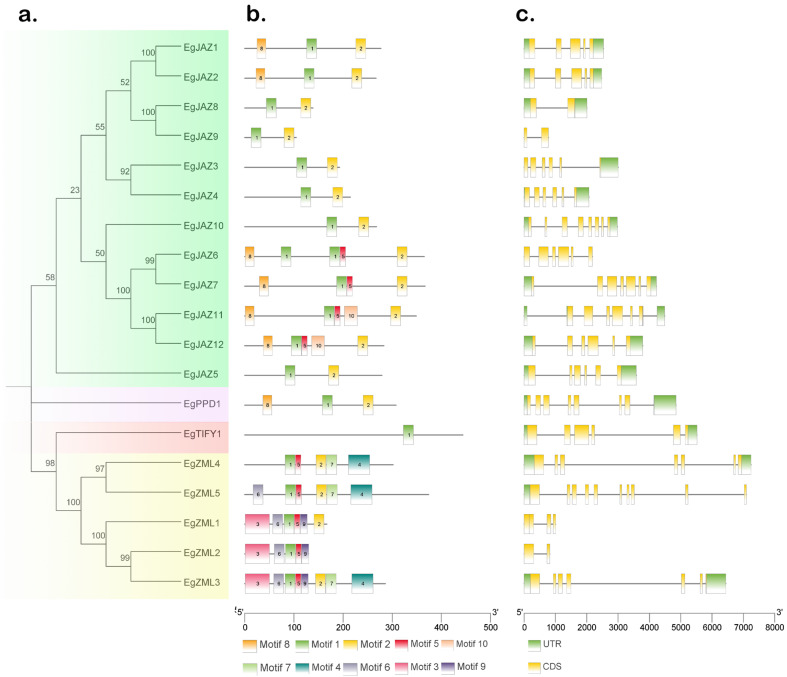
Analysis of conserved motifs and gene structure of *EgTIFYs*. (**a**) Neighbor-joining phylogenetic analysis (Bootstrap: 1000) of EgTIFYs proteins illustrating subfamily classifications, with four different colors representing four subfamilies: light green (JAZ), purple (PPD), red (TIFY), and yellow (ZML). (**b**) The different colored modules (Motif 1–Motif 10) represent the conserved amino acid sequence patterns in members of the gene family. (**c**) The intron and exon maps present the structural composition of the gene, with the yellow region representing the coding sequence (CDS) and the green region representing the untranslated region (UTR).

The 19 EgTIFY proteins were classified into four subfamilies according to conserved motifs and phylogenetic relationships (Figure 1). Conserved motifs were detected via the MEME suite, revealing 10 distinct motifs (Figure 2b). Motif 1 was conserved across all EgTIFY proteins, and 17 of the 19 genes contained Motif 2, with the exception of *EgZML2* and *EgTIFY1*. Gene structure analysis revealed that *EgTIFYs* contained 2–10 exons and 0–4 introns (Figure 2c). Among them, *EgZML5* had the most complex structure with 10 exons and 1 intron, while *EgJAZ9* and *EgZML2* had the simplest structure, each with only 2 exons and no introns.

### 2.4. Chromosomal Localization of EgTIFYs

**Figure 3:**
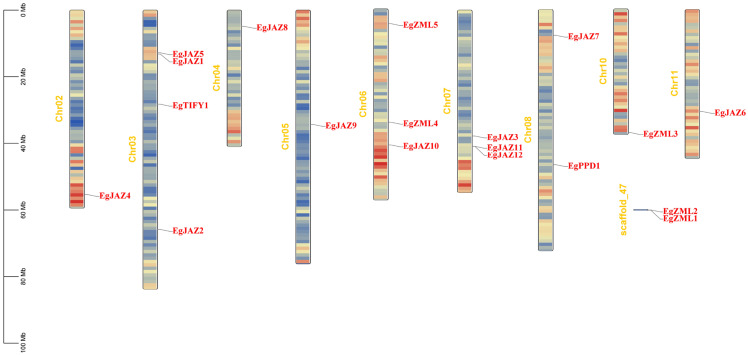
Chromosome identifiers are centrally positioned. Scale bars (Mb) and color gradients (red: high gene density; blue: low gene density) denote genomic distribution patterns.

Chromosomal mapping analysis indicated that the 19 *EgTIFY* genes are dispersed across nine chromosomes (chromosomes 2, 3, 4, 5, 6, 7, 8, 10, and 11; Figure 3). Chromosome 3 harbored the highest number of *EgTIFY* genes (four: *EgJAZ1*, *EgJAZ2*, *EgJAZ5*, and *EgTIFY1*), followed by chromosomes 6 and 7, each containing three genes. Chromosome 8 carried two genes, while the remaining chromosomes each contained one. Notably, *EgZML1* and *EgZML2* were localized to the same scaffold and not anchored to any chromosome. Additionally, the *EgTIFY* genes were dispersed across chromosomes without forming clusters.

### 2.5. Analysis of Intraspecific and Interspecific Collinearity of EgTIFY Genes

**Figure 4:**
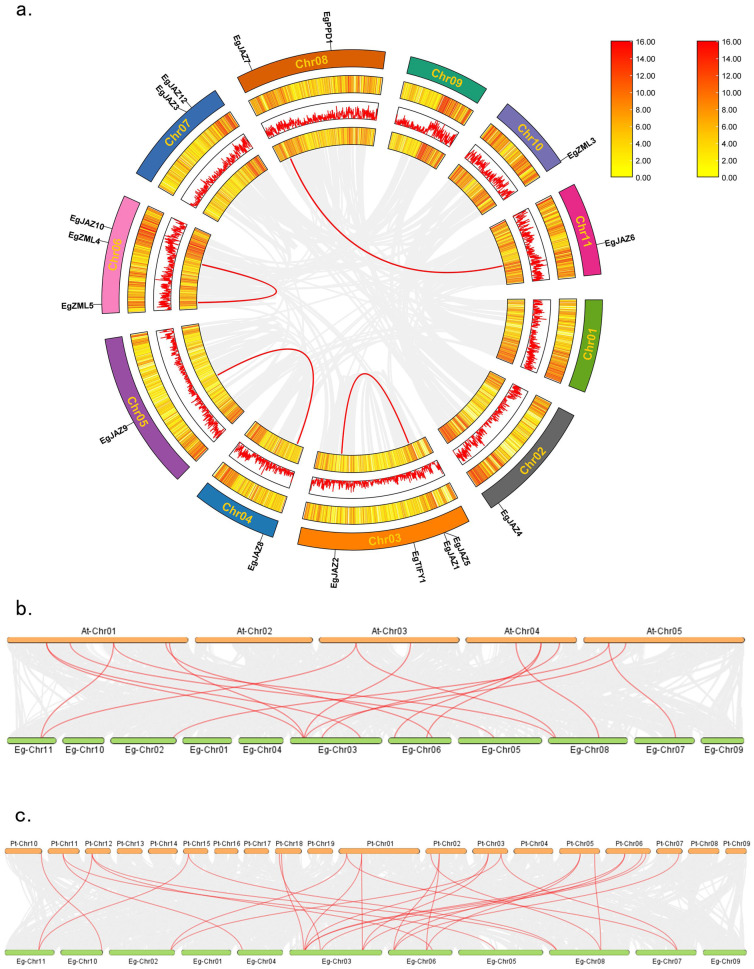
Collinearity profiles of *TIFY* family members across *E. grandis*, *P. trichocarpa*, and *A. thaliana*. Intraspecific collinear associations of *TIFY* family members in *E. grandis* (**a**). The three rings from inside to outside represent gene densities, presented as a heatmap and lines, respectively, and the rectangle in the upper right corner represents the gene density scale. The outermost ring represents the chromosome position. Gray lines represent all collinear blocks within the *E. grandis* genome, whereas red lines indicate segmental duplication events of *EgTIFY* genes. Additionally, interspecific collinear relationships are depicted between *E. grandis* and *A. thaliana* (**b**), and between *E. grandis* and *P. trichocarpa* (**c**). Red lines mark the duplication events of *TIFY* gene pairs.

To investigate whole-genome duplication events involving *EgTIFY* genes, an intra-specific synteny analysis was conducted for the *TIFY* gene family in *E. grandis* (Figure 4a). The findings indicated that the *TIFY* gene family in *E. grandis* has undergone infrequent intragenomic duplication events, with a mere four pairs of tandem and segmental duplications revealed: *EgJAZ6*-*EgJAZ7*, *EgJAZ2*-*EgJAZ1*/*EgJAZ5*, *EgJAZ8*-*EgJAZ9*, and *EgZML4*-*EgZML5*. In interspecific synteny analyses, 17 collinear gene pairs were identified between *E. grandis* and *A. thaliana*, whereas 29 gene pairs were found between *E. grandis* and *P. trichocarpa*, indicating a higher degree of evolutionary conservation between *E. grandis* and *P. trichocarpa*.

### 2.6. Analysis of cis-Acting Elements in the Promoters of EgTIFY Genes

**Figure 5:**
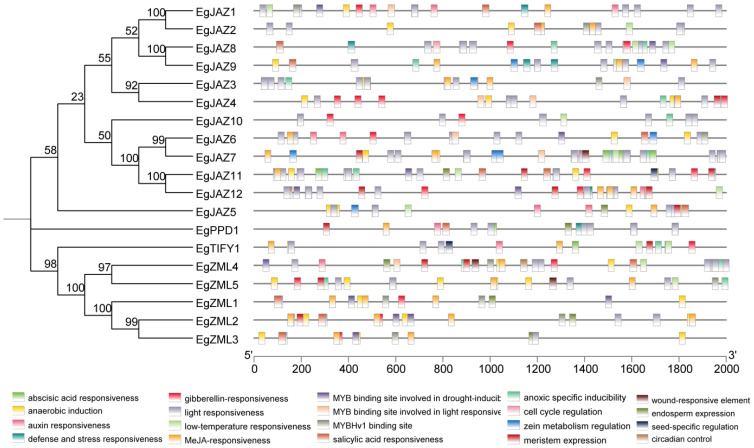
Prediction of cis-acting elements in the *EgTIFYs*. The 20 boxs on the lower side show the different cis-acting elements in the *TIFY* gene family promoters. The NJ tree was constructed 1000 bootstrap replicates.

To elucidate the regulatory mechanisms governing *EgTIFY* expression, 2 kb upstream promoter regions of all 19 genes were interrogated for cis-acting elements (Figure 5). Twenty representative elements were uncovered and categorized into four functional classes: development-associated, light-responsive, hormone-responsive, and stress-responsive. Hormone-responsive elements were the most abundant. For instance, *EgJAZ11* contained cis-elements responsive to abscisic acid, gibberellin, JA, salicylic acid, auxin, and multiple stress stimuli, suggesting a broad regulatory role in phytohormone signaling.

### 2.7. Expression Profiles of EgTIFY Genes in Diverse Tissues

**Figure 6:**
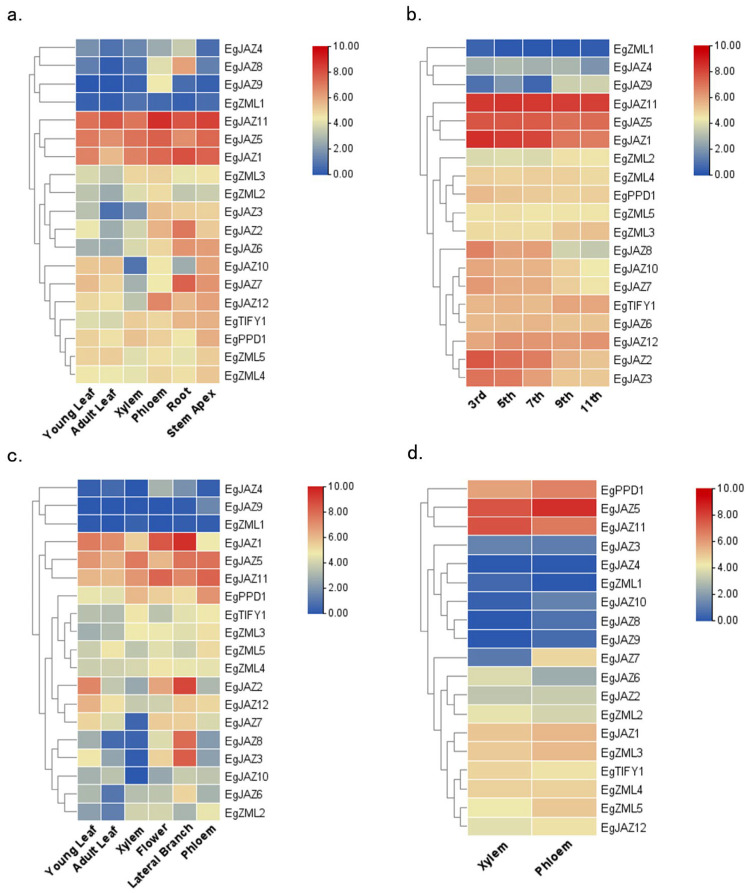
Tissue-specific expression heatmaps of *EgTIFY* genes. (**a**) Transcriptional profiling across juvenile tissues: 6-month-old leaves (young/adult), xylem, phloem, stem apex and roots (**b**). Differential expression analysis in mature stem internodes (3rd, 5th, 7th, 9th, and 11th) of adult trees. (**c**) Expression heatmaps in 3-year-old young leaves, adult leaves, xylem, flowers, lateral branches and phloem. (**d**) Expression heatmap of 6-month-old xylem and phloem tissues in *E. grandis*. RNA-seq-derived expression values are visualized using a color gradient from blue to red, reflecting low to high expression magnitudes, respectively.

To obtain a more profound understanding of the functional roles of *TIFY* genes in *E*. *grandis*, their expression patterns were examined using transcriptome data, and the accuracy of these expression levels was previously validated through quantitative real-time PCR experiments [24]. Expression levels of EgTIFY genes were evaluated across diverse tissues, including leaves, xylem, and phloem, of both 6-month-old and 3-year-old plants (Figure 6). The results revealed that *EgTIFY* gene expression is highly tissue- and stage-specific. Based on their expression patterns, the genes were categorized into three groups: constitutively expressed, lowly expressed, and stably expressed. Constitutively expressed genes, such as *EgJAZ1*, *EgJAZ5*, and *EgJAZ11*, exhibited consistently high expression across all tissues and developmental stages (Figure 6). In contrast, genes including *EgZML1*, *EgJAZ9*, and *EgJAZ4* exhibited low expression in all sampled tissues, suggesting limited or specialized roles under normal physiological conditions. Meanwhile, stably expressed genes like *EgZML3* and *EgZML4* maintained relatively constant expression levels between the two developmental stages, indicating a role in maintaining basic cellular functions (Figure 6a,c). Among all genes, *EgJAZ11* and *EgJAZ5* showed particularly high expression in xylem and phloem tissues, suggesting a possible role in regulating secondary cell wall formation and vascular development in *E. grandis* (Figure 6b). Conversely, the consistently low expression levels of *EgJAZ4*, *EgJAZ9*, and *EgZML1* across tissues may indicate a reduced functional demand or tightly regulated activity under standard growth conditions. Overall, these expression profiles provide meaningful elucidations regarding the tissue-specific and developmental roles of *EgTIFY* genes in *E. grandis* physiology.

### 2.8. Expression of EgTIFY Genes Under Abiotic Stress and Phytohormone Treatments

**Figure 7:**
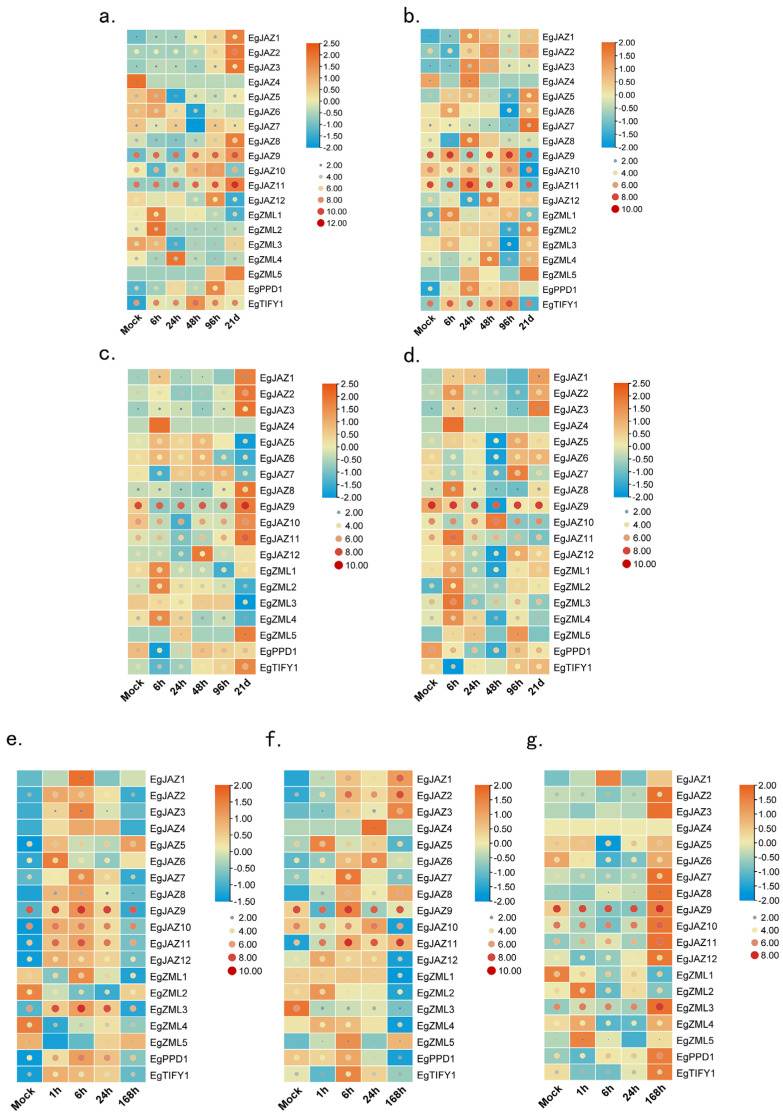
Gene expression of *EgTIFY* genes under abiotic stress and hormone treatments. (**a**–**d**) Expression pattern of *EgTIFY* genes under boron deficiency (**a**,**c**) and phosphorus deficiency treatment (**b**,**d**) after 0 h, 6 h, 24 h, 48 h, 96 h, and 21 d. The root (**a**,**b**) and stems (**c**,**d**) were used as samples. (**e**–**g**) Expression levels of *EgTIFY* genes at 0 h, 1 h, 6 h, 24 h, and 168 h under JA treatment (**e**), salt stress (**f**), and SA treatment (**g**). Transcript abundance is represented using a blue-to-red gradient, corresponding to log2-normalized RNA-Seq expression values (low to high). The color and size of the circles correlate with raw *EgTIFY* expression values: larger, redder circles reflect higher expression magnitudes, whereas smaller, bluer circles indicate lower magnitudes. Rectangular plots are row-normalized, with blue representing lower expression at each processing length and deeper orange indicating higher expression at the same length.

To examine the expression dynamics of the *EgTIFY* gene family under various abiotic stress and phytohormone treatments, two-month-old *E. grandis* seedlings were exposed to a set of experimental treatments, following the protocol described by Fan et al. (2024) [24]. These treatments included boron and phosphorus deficiency (applied to both roots and stems), foliar application of SA and JA, and exposure to salt stress. Gene expression profiles were monitored to delineate regulatory patterns in response to these stimuli. Circular visualization of raw expression data indicated that *EgJAZ9* was consistently upregulated across all treatments, highlighting its broad responsiveness to diverse environmental cues (Figure 7). Further analysis using row-normalized rectangular graphs revealed distinct temporal variations in gene expression patterns. For example, under boron deficiency, *EgJAZ1*, *EgJAZ2*, and *EgJAZ3* exhibited a long-term response, with expression peaking in both stems and roots after 21 days of treatment (Figure 7a,c). In contrast, *ZML* genes (*ZML1–5*) displayed a rapid response to phosphorus deficiency in stems, reaching maximal expression within 1 h. All genes in the JAZ cluster exhibited induced expression under JA, SA, and salt stress treatments, although their peak expression levels occurred at distinct time points (Figure 7e–g). Notably, *EgJAZ9*, which displays low basal expression under normal conditions, showed marked upregulation under these stress treatments, indicating strong induction by JA, SA, and salt stress (Figure 7e–g). Under JA and salt stress, several genes, including *EgJAZ7*, *EgJAZ12*, and *EgPPD1,* exhibited biphasic expression patterns, characterized by an initial induction phase followed by downregulation, indicative of a complex regulatory mechanism in response to these stimuli (Figure 7e,f). Under SA treatment, most *EgTIFY* genes showed progressive upregulation throughout the experimental period (Figure 7g).

### 2.9. Three-Dimensional Structure Analysis of E. grandis TIFY Gene Family Members

**Figure 8:**
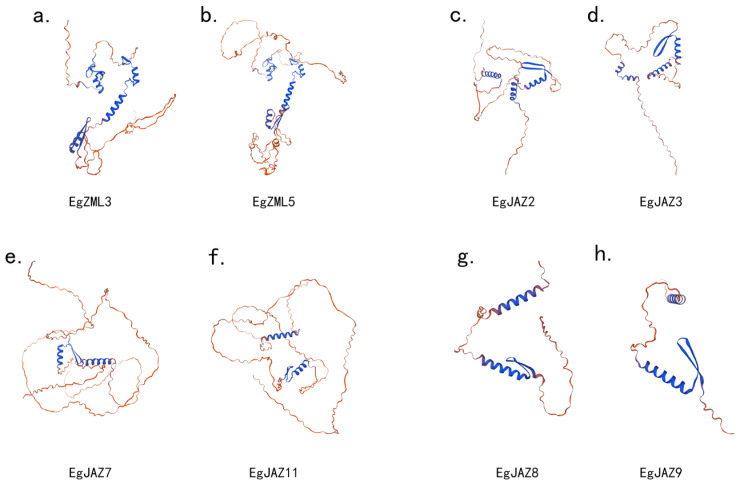
Three-dimensional structural analysis of *EgTIFY* gene family members. (**a**,**b**) Three-dimensional structure of EgZML3 and EgZML5, belonging to the ZML protein subfamily. (**c**,**d**) Three-dimensional structure of EgJAZ2 and EgJAZ3 from the JAZ protein subfamily. (**e**,**f**) Three-dimensional structure of EgJAZ7 and EgJAZ11 from the JAZ protein subfamily. (**g**,**h**) Three-dimensional structure of EgJAZ8 and EgJAZ9, member sof the JAZ protein subfamily.

In order to gain a deeper understanding of the structural features and functional diversity of the *EgTIFY* genes. The 3D architectures of proteins within identical subfamilies display striking structural conservation were performed (Figure 8). These structures were verified using Ramachandran plots (Supplementary Figure S1). In contrast, inter-subfamily comparisons reveal substantially enhanced structural divergence. This variation predominantly stems from disparities in the number and length of α-helical segments, β-turn configurations, and non-regular loop conformations unique to each protein. Such differential arrangements directly manifest as alterations in tertiary folding geometries, potentially accounting for the specialized functional capacities exhibited across these molecular entities [24].

## 3. Discussion

GATA transcription factors, characterized by their unique zinc finger structure, are widely distributed in eukaryotes and represent one of the most extensively studied families of transcription factors [25]. These molecules have been experimentally validated to govern a range of biological processes in plants, exemplified by seed germination, organ development, carbon and nitrogen metabolism, and stress responses [26]. The *TIFY* gene family is a plant-specific subfamily of GATA transcription factors, named for the conserved TIFY domain, which contains the amino acid sequence TIF[F/Y]XG (where X represents any amino acid). In this study, 19 *TIFY* gene family members were identified in *E. grandis*, and comprehensive analyses were conducted to investigate the evolutionary dynamics of this gene family. The number of TIFY members identified in *E. grandis* is comparable to those found in *Actinidia chinensis* [27] and the overall family structure is consistent with that of other dicotyledonous plants.

Predictions of subcellular localization demonstrated that all EgTIFY proteins are situated in the nucleus (Table 1). This finding carries notable biological implications, particularly in the context of the TIFY family’s functional conservation and species-specific adaptations. This also suggests that EgTIFYs likely exert their biological functions through direct involvement in nuclear transcriptional networks, potentially governing pathways critical to its growth as a woody species, such as xylem development, stress tolerance, or hormone-mediated responses. Nuclear localization aligns with the canonical role of TIFY proteins as transcriptional regulators, a function well-documented in various plant species [1]. TIFY family members, such as JAZ proteins, are known to modulate gene expression by interacting with transcription factors, hormone receptors, or other regulatory proteins within the nucleus, which is the key processes in stress signaling, growth, and development [28]. The exclusive nuclear localization of EgTIFYs exhibits both conservation and divergence compared to other plants. This result is consistent with findings in cucumber (*Cucumis sativus*), where all TIFYs were also reported to be nuclear-localized [11], pointing to a conserved functional paradigm in certain species. In contrast, TIFY proteins in other species, such as *P. trichocarpa* [29] and walnut [16], have been localized to additional subcellular compartments, including chloroplasts, mitochondria, and the endoplasmic reticulum. These differences may reflect species-specific adaptations: while some TIFYs in woody plants like poplar and walnut may have evolved to perform non-nuclear roles (e.g., organelle-specific stress responses or metabolic regulation), the strict nuclear confinement of *EgTIFYs* suggests a focused specialization in nuclear-mediated transcriptional regulation in *E. grandis*.

Physicochemical property analysis revealed significant variation among the 19 *EgTIFY* members (Table 1), which may reflect functional diversification during development or long-term environmental adaptation. Consistent with observations in maize, *Ricinus communis*, and tobacco, most GATA proteins were found to be structurally stable and hydrophilic [29,30]. Examination of conserved motifs and gene construction revealed that Motif 1 is shared among all *EgTIFY* transcription factors, suggesting it is a highly conserved feature within the *E. grandis* genome (Figure 2). Members within the same subfamily displayed analogous motif compositions, reflecting a high level of conservation and suggesting potential functional commonality. For example, Motif 3 was uniquely present in *EgZML1*, *EgZML2*, and *EgZML3* of the ZML subfamily, suggesting these genes may perform distinct functions in *E. grandis*.

Phylogenetic analysis divided the 19 *EgTIFYs* into four subfamilies: TIFY, ZML, JAZ and PPD. The JAZ subfamily was further partitioned into JAZ I and JAZ II subgroups (Figure 1) [31]. The phylogenetic topology of *E. grandis TIFY* genes closely resembled that of *A. thaliana* and *P. trichocarpa*, while *Physcomitrium patens* lacked members of the JAZ and PPD subfamilies [7,32,33]. This suggests gene loss events in the bryophyte lineage during evolution, whereas angiosperms such as *A. thaliana* and *P. trichocarpa* retained these genes, contributing to their more complex gene regulatory networks and enhanced adaptability. Gene family expansion in plants is generally driven by whole-genome duplication, tandem duplication and segmental duplication [34,35]. Comparative genomic investigations demonstrated that *TIFY* genes in *E. grandis* exhibit greater collinearity with those in *P. trichocarpa* than with *A. thaliana* (Figure 4b,c). This likely reflects that compared to the herbaceous *A. thaliana*, the two woody species share a more recent common ancestor, which probably accounts for this pattern.

Previous studies have established that cis-acting elements function as critical molecular switches in the transcriptional regulation of genes under abiotic or biotic stress [29]. Analysis of *EgTIFY* promoter regions identified four classes of hormone-responsive cis-elements, specifically those responsive to auxin, JA, gibberellin, and SA. Within the JAZ subclade (consisting of 12 genes), all members displayed rapid induction following JA treatment, with significant upregulation detectable as early as 1 h post-application. Additionally, these genes exhibited a delayed yet sustained response to SA, with marked upregulation observed after 7 days of treatment (Figure 7). Promoter analysis further revealed that most *JAZ* genes contain JA-responsive cis-elements, except for *JAZ8* and *JAZ10*; SA-responsive cis-elements were absent in *JAZ3*, *JAZ10*, *JAZ12*, and *JAZ7* (Figure 5). These findings suggest that the majority of this JAZ subclade may directly respond to JA and SA signals to coordinate the expression of downstream genes involved in plant growth, development, and defense responses. Previous research has highlighted the pivotal role of MYB transcription factors in regulating plant responses to boron deficiency. For instance, MYB transcription factors in *A. thaliana* have been implicated to play functional roles under low-boron conditions, mediating adaptation to boron stress via transcriptional reprogramming [36]. In our study, *EgZML1* to *EgZML4* exhibited a relatively strong and early transcriptional response, with induction detected 1 h after the initiation of boron deficiency treatment (Figure 7a,c). Promoter analysis indicated that these four *ZML* genes each harbor three MYB-binding cis-elements, suggesting potential transcriptional regulation by MYB factors under boron-deficient conditions. In contrast, *ZML5*, which lacks MYB-binding sites in its promoter, did not exhibit a similar expression pattern under the same treatment (Figure 5). These observations suggest that the differential responsiveness of *ZML* genes to boron deficiency may be mediated, at least in part, by the presence or absence of MYB regulatory elements. The number of these cis-elements varied among *EgTIFY* genes, indicating differential hormone responsiveness and potential functional divergence. Additionally, abiotic stress-related cis-elements, including those responsive to light and low temperature, were detected, suggesting that *EgTIFY* genes are involved in defense and stress response pathways (Figure 5).

The high economic value of *Eucalyptus* wood has spurred extensive research aimed at elucidating the genetic mechanisms underlying wood formation, with the objective of improving both wood yield and quality through genetic interventions [37]. Wood formation constitutes a intricate, protracted biological process encompassing orchestrated events including cell division, cellular differentiation, secondary cell wall (SCW) biosynthesis, and programmed cell death (PCD) [38]. Secondary growth is primarily regulated by the vascular cambium and includes the formation of vascular tissues, SCW biosynthesis, lignification, heartwood formation, and PCD. The biosynthesis of SCWs relies on the organized production and assembly of four key components: cellulose and hemicellulose as structural polysaccharides, lignin, structural proteins, and specific secondary metabolites, including flavonoids, tannins, and pectins. Among these, the highly ordered deposition of cellulose microfibrils and lignin is critical for conferring strength and rigidity to the developing xylem tissue [4,5]. JAZ proteins, serving as core constituents of the jasmonic acid signaling pathway, have been demonstrated to participate in a wide array of plant developmental processes. Previous research has established that *JAZ* genes display tissue-specific expression profiles and fulfill crucial roles in plant development and stress responses. For instance, real-time quantitative PCR (qRT-PCR) analysis revealed that seven *CsJAZ* genes were preferentially expressed in the roots of tea plants [39]. They act by interacting with a wide variety of proteins, including transcription factors, signaling regulators, and pathogen effectors, to modulate biological outcomes such as anthocyanin accumulation, trichome initiation [24,25], fiber elongation in cotton [40], stamen differentiation [41], flowering time regulation [42,43], pathogen defense [21,44,45], insect resistance [46], and responses to abiotic stresses [47,48]. To better understand the functional roles of *TIFY* genes in *E. grandis*, their expression profiles across various tissues and under hormone treatments using transcriptomic data were analyzed (Figure 6 and Figure 7). The reliability of these expression patterns was previously validated through qRT-PCR [24]. *EgJAZ1*, *EgJAZ5*, and *EgJAZ11* were found to be constitutively expressed at high levels across all tissues, particularly in stem nodes. These genes also maintained strong expression in both 6-month-old and 3-year-old *E. grandis* individuals (Figure 6). Phylogenetic and sequence similarity analyses indicated that *EgJAZ5* is orthologous to *PtJAZ6*, sharing conserved motifs and a close evolutionary relationship (Figure 1). Significantly, *PtJAZ6* exhibits preferential expression in roots and vessels and has been functionally linked to pest response, suggesting that *EgJAZ5* may possess analogous biological roles [49]. In contrast, *EgZML1* exhibited consistently low expression across tissues, while *EgJAZ2* and *EgJAZ7* were predominantly expressed in roots, indicating tissue-specific expression and potential involvement in root development or stress responses compared with other species. Based on these observations, it is plausible that *EgJAZ5* may play a similar role in *E. grandis*, contributing to vascular development and cell wall biosynthesis through JA-dependent regulatory mechanisms.

JA, a key regulatory phytohormone involved in secondary metabolism, is widely recognized for its ability to induce the biosynthesis of flavonoids and other specialized metabolites [50]. Within the *TIFY* gene family, the JAZ subfamily is the most extensively characterized, acting as central repressors in the JA signaling pathway. JAZ proteins act as mediators of plant responses to diverse abiotic stressors, including drought, osmotic stress, salinity, and heavy metal toxicity [51]. In the absence of active JA signaling, JAZ proteins inhibit transcription factors such as MYC and MYB by forming complexes via their conserved ZIM and Jas domains, thereby repressing the transcription of downstream stress-responsive genes [52]. Moreover, members of the *TIFY* family are also implicated in the regulation of terpenoid biosynthesis, often through interactions with various transcription factors in a hormone-dependent manner [53,54]. Several studies have demonstrated that JA can significantly induce *JAZ* gene expression. For example, in *Prunus persica*, *PpJAZ1*, *PpJAZ4*, *PpJAZ5*, and *PpJAZ7* were markedly upregulated following treatment with 200 μmol·L^−1^ JA [39]. In *Solanum melongena*, JA-induced expression of *SmMYB5* was shown to activate a suite of flavonoid biosynthesis genes, including *SmCHS*, *SmF3H*, *SmDFR*, and *SmANS*, resulting in enhanced flavonoid accumulation [55]. In our study, the promoter region of *EgJAZ9* harbors multiple cis-acting elements linked to light responsiveness, JA and ABA sensitivity, alongside a MYB-binding site implicated in drought induction (Figure 5). Phylogenetic analysis identified two close homologs of *EgJAZ9*, one in *P. trichocarpa* and another in *A. thaliana* (Figure 1). Previous studies have shown that *PtJAZ9* participates in complex regulatory networks related to pest-induced and cold stress, which is also highly responsive to JA signaling [49]. Additionally, in *Arabidopsis*, mutation of *JAZ7* under dark conditions leads to the release of MYC2/3/4 transcription factors, which bind to G-box motifs in target promoters and activate genes involved in indole-glucosinolate biosynthesis, sulfur metabolism, callose deposition, and JA signaling, thereby promoting dark-induced leaf senescence [56]. Based on these findings and conserved regulatory features, it is plausible that *EgJAZ9* may play a central role in JA-mediated signaling and associated physiological processes in *E. grandis*.

The three-dimensional structure of the EgTIFY protein (Figure 8) highlights a notable abundance of loop regions, primarily attributed to the intrinsic structural disorder within its key functional domains, particularly the Jas domain. This feature is characteristic of the TIFY/JAZ protein family and is essential for their regulatory flexibility [57]. In *A. thaliana*, the functional jasmonate receptor is formed by a complex between COI1 and JAZ proteins [58]. COI1 contains an open binding pocket that specifically recognizes the bioactive jasmonate hormone, (3R,7S)-jasmonoyl-L-isoleucine (JA-Ile). High-affinity binding of JA-Ile requires a bipartite degron motif within the JAZ protein, composed of a conserved α-helix that docks onto COI1 and an adjacent flexible loop region. This loop plays a critical role in stabilizing the hormone within the COI1 pocket, effectively facilitating hormone perception and subsequent signaling [59]. The prevalence of such disordered regions in *EgTIFY* proteins may similarly contribute to their structural adaptability and functional interaction with hormonal signaling components in *E. grandis* [60].

In conclusion, *TIFY* family members in *E. grandis* exhibit tissue-specific expression patterns at various developmental stages, suggesting functional divergence likely driven by gene mutations and evolutionary pressures. These findings lay the foundation for future functional validation and the elucidation of *TIFY* gene regulatory networks. However, the function of these *TIFY* genes should be further examined by experiments.

## 4. Materials and Methods

### 4.1. Identification of EgTIFYs Members and Analysis of Physicochemical Properties

Nucleotide sequences encoding *TIFY* family members from *E*. *grandis* and *A*. *thaliana* were obtained from the Phytozome database (https://phytozome-next.jgi.doe.gov/, accessed on 19 February 2025) and TAIR database (http://www.arabidopsis.org, accessed on 19 February 2025), respectively. These genomic sequences were translated into protein sequences using TBtools-II software (version 1.120) [61]. A total of 19 TIFY family members were identified through comparative sequence alignment of TIFY proteins from *A. thaliana* and *E. grandis*, utilizing the “Blast Compare Two Seqs” function in TBtools-II (accessed on 19 February 2025) with an E-value cutoff set at 10^−5^. Physicochemical properties of *E. grandis* TIFY proteins, such as molecular mass, isoelectric point, and amino acid count, were analyzed via the ProtParam tool available on the Expasy database (https://www.expasy.org/, accessed on 19 February 2025). Subcellular localization of EgTIFY proteins was predicted using the WoLFPSORT program (https://www.genscript.com/wolf-psort.html, accessed on 19 February 2025).

### 4.2. Phylogenetic Analysis of the EgTIFYs

*TIFYs* from *A. thaliana, P. patens* and *P. trichocarpa* were used to perform comparative sequence analysis and similarity assessment. *A. thaliana* genome annotation was obtained from the TAIR (https://www.arabidopsis.org/, accessed on 20 February 2025). Genome annotation information of *P. patens* and *P. trichocarpa* was derived from Ensembl Plants (https://plants.ensembl.org/index.html, accessed on 20 February 2025). Evolutionary analyses of the three species were performed using MEGA11, constructed by the neighbor-joining (NJ) method (Bootstrap: 1000) [62]. The evolutionary tree was then categorized and annotated using the evolview (http://www.evolgenius.info/evolview, accessed on 20 February 2025) website.

### 4.3. Analysis Gene Structures, Conserved Motifs and Conserved Domains of EgTIFYs

The MEME (https://meme-suite.org/meme/, accessed on 20 February 2025) was used to predict the conserved motifs of *EgTIFYs*, and their conserved structural domains were analyzed by NCBI-CDD (https://www.ncbi.nlm.nih.gov/cdd/, accessed on 20 February 2025), and finally visualized and analyzed using TBtools-II software.

### 4.4. Gene Family Chromosomal Distribution and Synteny Analysis

To determine the chromosomal distribution of the 19 *E. grandis TIFY* genes, the “Gene Location Visualize from GTF/GFF” function in TBtools-II software was used. This tool allowed mapping of each gene to its corresponding physical position on the chromosomes. To further assess the genomic distribution, a gene density heatmap was generated using the “Gene Density Profile” module with a bin size set at 100,000 bp, while maintaining all other parameters at default settings. For collinearity and synteny analysis, gene annotation and sequence files from *E. grandis*, *P. alba* and *A. thaliana* were imported into the “One Step MCScanX” module within TBtools-II software. Syntenic relationships between *E. grandis* and the two reference species were illustrated using the “Dual Synteny Plot” function. To explore internal collinearity within the *E. grandis* genome, a genome-wide synteny analysis was also performed using “One Step MCScanX,” and the results were visualized through the “Advanced Circos” module. In the MCScanX analysis, parameters were configured with a BLASTP CPU thread count of 2, an E-value threshold of 10^−10^, and the top 5 BLAST hits retained for downstream synteny detection.

### 4.5. Analysis of Promoter cis-Regulatory Element of the EgTIFYs

The promoter sequence of the first 2000 bp upstream of the initiator of *TIFY* family members was extracted, and its cis-acting elements were predicted using the online website PlantCARE (https://bioinformatics.psb.ugent.be/webtools/plantcare/html/, accessed on 25 February 2025), and finally visualized using TBtools-II software.

### 4.6. Expression Patterns of EgTIFYs

To examine the tissue-specific expression patterns of *E*. *grandis TIFY* genes, as well as the expression profiles under abiotic stress (boron deficiency, phosphorus deficiency, salt stress) and hormone treatments (JA, SA), transcriptome data from our previous study were reanalyzed [24]. Each biological replicate comprised tissues sampled from no fewer than three individual plants, with three technical replicates processed per measurement to ensure data reliability. Tissue dissection was performed manually. For stem-derived samples, longitudinal sections were first made to expose the epidermis. The outer phloem was gently removed using fine-tipped forceps, followed by precise scraping of the underlying xylem layer for collection. Expression quantification data were normalized and transformed using log_2_ scale prior to visualization. Hierarchical clustering based on Euclidean distance and complete linkage was performed in TBtools-II software to produce heatmaps, enabling the comparative assessment of transcriptional variation across stress conditions and tissue types. This approach facilitated the identification of distinct expression trends among the *TIFY* genes [24].

### 4.7. Protein Structure Prediction of EgTIFYs

Three-dimensional structures of EgTIFY proteins were predicted via the SWISS-MODEL platform (https://swissmodel.expasy.org/, accessed on 28 February 2025). Amino acid sequences were uploaded to the website, and models with the highest matching levels were selected—all chosen models showed over 50% similarity. The corresponding files were then exported for further analysis, with similarity verified using Ramachandran plots.

## 5. Conclusions

In this study, 19 members of the *TIFY* gene family were identified for the first time at the genome-wide level in *E. grandis*, and their potential involvement in hormone signaling pathways was predicted based on bioinformatics and expression analyses. Through promoter cis-acting element analysis, the promoter regions of several *EgTIFY* genes were found to contain gibberellin (GARE-motif), growth hormone (AuxRR-core), JA (CGTCA-motif), and salicylic acid (TCA-element) response elements, indicating that the genes of this family may participate in the growth, development, and stress response of *E. grandis* through hormone-mediated signaling pathways. The expression of JAZ subfamily member *EgJAZ9* was significantly up-regulated after JA treatment, and the Jas structural domains of the protein sequences were highly conserved with the *Arabidopsis* JAZ proteins, which suggests that these genes are important for jasmonate signaling, of which *EgJAZ9* could be a candidate gene for further study.

## Figures and Tables

**Table 1:** Biophysical characteristics of TIFY family proteins in *E. grandis*.

Name	Sequence ID	Number of Amino Acids	Molecular Weight	Theoretical pI	Instability Index	Aliphatic Index	Grand Average of Hydropathicity	Subcellular Localization
EgJAZ1	Eucgr.C00785.1.v2.0	276.00	28,841.80	9.30	59.83	66.67	−0.33	nucleus
EgJAZ2	Eucgr.C03301.1.v2.0	266.00	27,833.55	9.49	62.68	63.76	−0.43	nucleus
EgJAZ3	Eucgr.G01954.1.v2.0	192.00	20,318.31	10.55	81.53	73.33	−0.43	nucleus
EgJAZ4	Eucgr.B03545.1.v2.0	214.00	23,686.17	9.67	58.22	66.59	−0.45	nucleus
EgJAZ5	Eucgr.C00753.1.v2.0	278.00	29,805.96	6.34	54.01	83.27	−0.35	nucleus
EgJAZ6	Eucgr.K02279.1.v2.0	364.00	38,714.78	8.57	58.70	72.86	−0.34	nucleus
EgJAZ7	Eucgr.H00537.1.v2.0	366.00	38,860.62	9.80	60.88	69.15	−0.41	nucleus
EgJAZ8	Eucgr.D00289.1.v2.0	138.00	15,408.21	9.26	99.17	60.14	−0.75	nucleus
EgJAZ9	Eucgr.E02416.1.v2.0	104.00	11,944.55	9.44	84.50	63.75	−0.69	nucleus
EgJAZ10	Eucgr.F02865.1.v2.0	267.00	30,050.97	9.40	61.95	69.10	−0.69	nucleus
EgJAZ11	Eucgr.G02115.2.v2.0	348.00	37,051.06	9.80	54.90	67.84	−0.38	nucleus
EgJAZ12	Eucgr.G02116.1.v2.0	282.00	29,576.68	9.09	52.04	72.34	−0.31	nucleus
EgPPD1	Eucgr.H03414.1.v2.0	307.00	33,447.75	8.86	44.15	64.59	−0.68	nucleus
EgTIFY1	Eucgr.C01809.1.v2.0	443.00	46,526.69	9.46	55.44	58.58	−0.65	nucleus
EgZML1	Eucgr.L00755.1.v2.0	166.00	18,189.11	6.22	31.19	31.19	−0.80	nucleus
EgZML2	Eucgr.L00751.1.v2.0	130.00	13,565.47	4.41	31.93	57.69	−0.89	nucleus
EgZML3	Eucgr.J03166.1.v2.0	285.00	31,048.07	6.04	38.27	54.07	−0.99	nucleus
EgZML4	Eucgr.F02157.1.v2.0	301.00	32,224.92	6.45	40.06	58.37	−0.61	nucleus
EgZML5	Eucgr.F00433.1.v2.0	373.00	40,095.13	4.87	41.57	64.34	−0.68	nucleus

## Data Availability

The datasets presented in this study can be found in online repositories. The names of the repository/repositories and accession number(s) can be found in the article/Supplementary Materials.

## Supplementary Materials

The following supporting information can be downloaded at https://www.mdpi.com/article/10.3390/ijms26167914/s1.

## Abbreviations

GlcA: glucuronic acid; Nucl: nucleus; NJ: Neighbor-Joining Algorithm; JA: Methyl Jasmonate; JA-Ile: jasmonoyl-L-isoleucine; SA: salicylic acid; TIFY: highly conserved structural domain (TIF[F/Y]XG); bHLH: helix–loop–helix; *E. grandis: Eucalyptus grandis; A. thaliana: Arabidopsis thaliana; P. trichocarpa: Populus trichocarpa; P. patens: Physcomitrella patens.*
