# Supplementary material for: Genome-Wide Identification and Expressional Analysis of the TIFY Gene Family in Eucalyptus grandis
Source: Int J Mol Sci. 2025 Aug 16;26(16):7914. doi: 10.3390/ijms26167914 (PMC12386639; doi:10.3390/ijms26167914)
Supplement: Supplementary file 1 [file ijms-26-07914-s001.zip › Supplementary Table.pdf]

Table S1 Mapping table of gene name and gene ID

Scientific name	Accession number	Phylogenetic tree name
	NP_001319041.1	AtJAZ1
	NP_565096.1	AtJAZ2
	NP_566590.1	AtJAZ3
	NP_175283.2	AtJAZ4
	NP_564019.1	AtJAZ5
	NP_565043.1	AtJAZ6
	NP_181007.1	AtJAZ7
	NP_564349.1	AtJAZ8
	NP_001320988.1	AtJAZ9
Arabidopsis thaliana	NP_001330994.1	AtJAZ10
	NP_189930.1	AtJAZ11
	NP_197590.1	AtJAZ12
	NP_001078200.1	AtJAZ13
	NP_567442.2	AtPPD1
	NP_001329427.1	AtPPD2
	NP_567898.1	AtTIFY8
	NP_001190821.1	AtZIM
	NP_566676.1	AtZML1
	NP_564593.1	AtZML2
	XP_024383719.1	PpTIFY1
	XP_024356667.1	PpTIFY2
	XP_024360727.1	PpTIFY3
	XP_024399112.1	PpTIFY4
	XP_024376421.1	PpTIFY5
	XP_024378255.1	PpTIFY6
Physcomitrium patens	XP_024376075.1	PpTIFY7
	XP_024378251.1	PpTIFY8
	XP_024365843.1	PpTIFY9
	XP_024399346.1	PpTIFY10
	XP_024395439.1	PpZML1
	XP_024400206.1	PpZML2
	XP_024380115.1	PpZML3
	XP_024368631.1	PpZML4
	XP_024456030.2	PtJAZ1
	XP_006369119.3	PtJAZ2
	XP_002304118.3	PtJAZ3
	XP_002304653.3	PtJAZ4
Populus trichocarpa	XP_006381595.2	PtJAZ5
	XP_024460186.1	PtJAZ6
	XP_024463469.1	PtJAZ7
	XP_002314793.1	PtJAZ8

Populus trichocarpa	XP_002316712.1	PtJAZ9
	XP_006376709.2	PtJAZ10
	XP_024442808.1	PtJAZ11
	XP_002324786.1	PtJAZ12
	XP_002307919.3	PtJAZ13
	XP_006386257.1	PtPPD1
	XP_006383674.1	PtPPD2
	XP_024460135.1	PtTIFY1
	XP_002324269.3	PtTIFY2
	XP_002302363.3	PtZML1
	XP_002302364.2	PtZML2
	XP_002307331.2	PtZML3
	XP_006383573.2	PtZML4
	XP_052310415.1	PtZML5
	XP_006380338.2	PtZML6
	XP_002316441.2	PtZML7
	XP_024445181.1	PtZML8
