# Supplementary figures and images for: Genome-Wide Identification and Expressional Analysis of the TIFY Gene Family in Eucalyptus grandis
Source: Int J Mol Sci. 2025 Aug 16;26(16):7914. doi: 10.3390/ijms26167914 (PMC12386639; doi:10.3390/ijms26167914)

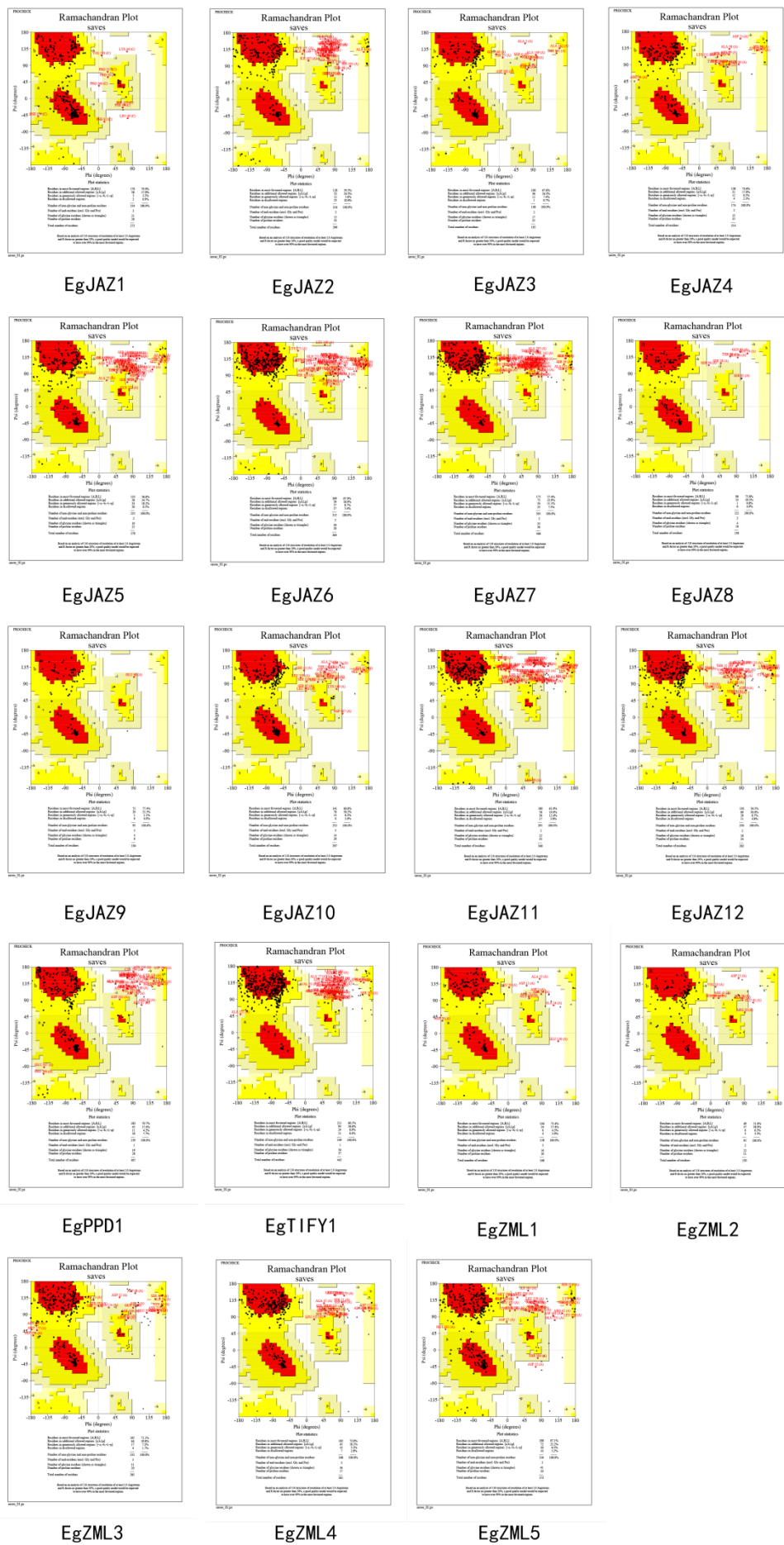

**Figure S1.** Ramachandran plot of *EgTIFY* gene family members.

Supplement: Supplementary file 1 [file ijms-26-07914-s001.zip › Supplementary Figure.pdf]
